# Parental separation in childhood as a risk factor for depression in adulthood: a community-based study of adolescents screened for depression and followed up after 15 years

**DOI:** 10.1186/s12888-017-1252-z

**Published:** 2017-03-29

**Authors:** Hannes Bohman, Sara Brolin Låftman, Aivar Päären, Ulf Jonsson

**Affiliations:** 1 Department of Neuroscience, Child and Adolescent Psychiatry, Uppsala University, Box 593, SE-75124 Uppsala, Sweden; 2 Department of Women’s and Children’s Health, Akademiska sjukhuset, SE-75185 Uppsala, Sweden; 3 Centre for Psychiatry Research, Department of Clinical Neuroscience, Karolinska Institutet, SE-17177 Stockholm, Sweden; 4 Stockholm Health Care Services, Stockholm County Council, Stockholm, Sweden; 5 Centre for Health Equity Studies (CHESS), Stockholm University/Karolinska Institutet, SE-10691 Stockholm, Sweden

**Keywords:** Adolescence, Divorce, Family type, Family structure, Depression, Prospective, Bipolar

## Abstract

**Background:**

Earlier research has investigated the association between parental separation and long-term health outcomes among offspring, but few studies have assessed the potentially moderating role of mental health status in adolescence. The aim of this study was to analyze whether parental separation in childhood predicts depression in adulthood and whether the pattern differs between individuals with and without earlier depression.

**Methods:**

A community-based sample of individuals with adolescent depression in 1991–93 and matched non-depressed peers were followed up using a structured diagnostic interview after 15 years. The participation rate was 65% (depressed *n* = 227; non-depressed controls *n* = 155). Information on parental separation and conditions in childhood and adolescence was collected at baseline. The outcome was depression between the ages 19–31 years; information on depression was collected at the follow-up diagnostic interview. The statistical method used was binary logistic regression.

**Results:**

Our analyses showed that depressed adolescents with separated parents had an excess risk of recurrence of depression in adulthood, compared with depressed adolescents with non-separated parents. In addition, among adolescents with depression, parental separation was associated with an increased risk of a switch to bipolar disorder in adulthood. Among the matched non-depressed peers, no associations between parental separation and adult depression or bipolar disorder were found.

**Conclusions:**

Parental separation may have long-lasting health consequences for vulnerable individuals who suffer from mental illness already in adolescence.

## Background

Numerous cross-sectional studies have shown that children who do not live with two original parents in the same household report poorer mental health outcomes compared with their peers in nuclear families [1–3], although the overall effect sizes are rather small [4]. Prospective studies of the association between parental separation in childhood and later mental health have demonstrated that individuals with separated parents are more likely to suffer from adverse mental health outcomes in adulthood [5–10]. There are however also studies that do not show such a relationship. One Swedish prospective study demonstrated that individuals with divorced parents were more likely to appear in child and adolescent psychiatric care compared with their peers with non-divorced parents, but that there was no difference between the groups with regard to adult psychiatric care [11]. Another study by the same authors did not find any overall differences in depression and anxiety in adulthood between individuals whose parents had divorced in childhood and the comparison group of individuals with continuously married parents [12]. Thus, empirical findings concerning parental divorce in childhood and later mental health outcomes are not fully consistent. As underscored in a review by Hetherington and Stanley-Hagan [13], however, the effects of parental divorce in childhood may differ between individuals. Some children are more vulnerable while others are more resilient, where resilience is thought to be a function of several factors including the characteristics of the child, the family, and the surrounding environment. As a matter of fact, Di Manno et al. [14] call for more studies on moderating effects of parental divorce and offspring mental health and claim that “investigations of moderation are in their infancy and further research is needed to understand the varying trajectories of mental health outcomes, specifically depression and depressive symptoms, of children of divorced parents.” (Di Manno et al. [14], p. 78). We argue that one potentially important vulnerability factor to consider is whether the child suffers or has previously suffered from mental disorders like depression. Depressive disorders are common in adolescence with a one-year prevalence of 5–8% [15]. Depressive disorders are also a major contributor to the global burden of disease [16], and among 15- to 19-year-old males and females, depression is the most important cause of disability-adjusted life-years (DALYs) [17] and thus of major public health concern.

Any association between parental separation and poorer mental health (short- and long-term) among offspring could be caused by several mechanisms, including factors both preceding and following the actual separation. One potential mechanism is inter-parental conflict, and indeed, Olsson [15] demonstrated that the association between parental separation and adolescent depression was accounted for by inter-parental conflict. Analyses of large-scale survey data have shown that severe dissension in the childhood family is more commonly reported by individuals who experienced their parents’ separation than by those who grew up in nuclear families, and that it contributes to explaining the adverse mental health of the former group [18]. In addition, studies have shown that separated parents are more likely to have poorer relations with their children compared with those who are continuously married, supposedly as an effect of the stress linked with the separation as such or with single parenthood [4]. Poorer parental relations, in turn, have been linked to poorer mental health among offspring [19]. Furthermore, parental separation often involves a loss of resources for children, particularly for those who continue to live with only one parent. Accordingly, absolute poverty in terms of low income standard is more common among children in single-parent households than among those in two-parent families [20]. Studies have shown that economic strain following a divorce is linked to adverse outcomes among children [4]. Furthermore, parental separation can lead to residential moves, which for the children may entail entering a new school and having to make new friends in an already stressful situation [13]. Indeed, changing schools has been shown to be linked with an increased risk of adverse mental health [21]. Unsurprisingly, studies have shown that it is more common for individuals with separated parents to have moved during childhood [12, 18]. Finally, the parents’ mental health should be considered. Marital distress and depression frequently co-occur, and it has been reported that the interaction of couples with a depressed partner is characterized by a higher frequency of negative communication and a lower frequency of positive communication [22]. Given that people with depression are more likely to divorce [23], and that parental psychopathology is a risk factor for mental disorders among offspring [24], any association between divorce and depression among offspring may partly be attributable to the heritability of depression. In addition, adults who have divorced tend to report poorer well-being compared with those who are continuously married [4]. This may negatively affect their ability to provide various kinds of social support to their children, with potentially negative implications for their children’s well-being. To conclude, as pointed out in the recent review by Di Manno et al. [14], it is important to investigate different types of moderating effects of parental separation on later depression. In the present study we focus on adolescent depression as a potential moderator.

### Aim of the study

The aim of the present study was to analyze whether the experience of parental separation in childhood predicts major depression in adulthood; and more specifically whether the association between parental separation and later major depression differs among individuals with and without adolescent depression. To assess the extent to which any possible associations were accounted for by potential covariates, we adjusted for conflicts between and with parents, economic strain, family moves, and parental depression.

We formulated the following hypotheses:

- H1. The experience of parental separation predicts adult depression.
- H2. The association between the experience of parental separation in childhood and depression in adulthood is more prominent among individuals who had suffered from adolescent depression than among individuals without adolescent depression.
- H3. The association between the experience of parental separation and adult depression is (at least partly) accounted for by major conflicts between parents, major conflicts with parents, economic strain, family moves, and parental depression.

## Methods

### Study population and procedure

The data come from a study of adolescent depression conducted in the town of Uppsala, Sweden, in 1991–1992. The purpose of the project was to investigate the prevalence of depression in a certain population and set up a case–control study based on a screening of a depression. Accordingly, all students in the first year of upper secondary school (ages 16–17 years) and school dropouts of the same age group were invited to participate in a screening for depression. Of 2,465 individuals, 2,300 (93%) participated in the screening. Two self-evaluations of depression were used: the Beck Depression Inventory-Child [25] and the Centre for Epidemiological Studies-Depression Scale for Children [26]. Adolescents with high scores (BDI-C ≥ 16 or CES-DC ≥ 30) or who reported a suicide attempt were interviewed diagnostically using the revised adolescent version of the Diagnostic Interview for Children and Adolescents (DICA-R-A) according to the DSM-III-R criteria [27]. From individuals with scores under the cut-off (i.e. BDI-C < 16 and CES-DC < 30), a control group was created from an equal number of peers who were matched for sex, age, and school class, and was also diagnostically interviewed in the same manner, using the DICA-R-A. In total, 307 adolescents with depressive symptoms and 302 non-depressed controls were interviewed and consented to be contacted for follow-up. All interviews were conducted face to face by in total six persons (specialists in child psychiatry and psychology students) [28]. Comorbidity was common among the depressed (87% had also other diagnoses than depression, the most common ones being anxiety disorders, specific phobia, and conduct disorders) but less common among the non-depressed (33% had a diagnosis, the most common diagnosis being specific phobia) [29].

The participants also completed the Children’s Life Events Inventory [30], which includes questions on life events related to the individual’s family and social situation. More information on the original baseline study, including characteristics of participants and of those who did not participate, can be found elsewhere [15, 31].

After 15 years, the depressed and non-depressed adolescents who had participated in the diagnostic interview as well as consented to participate in a follow-up study were contacted for a follow-up evaluation. This evaluation included the structured diagnostic interview Mini International Neuropsychiatric Interview Plus (M.I.N.I.) [32]. The follow-up interviews were conducted by a clinical psychologist, a psychiatrist, and three students in clinical psychology, who did not know whether the participants belonged to the depressed or the non-depressed control group (free-marginal Kappa of 0.93). A total of 409 of the 609 participants were re-interviewed (67%). Of all interviews, 81% were conducted face to face and 19% by telephone. For more information on the follow-up study, including details on participants and reasons for non-participation, see Jonsson et al. [33].

**Fig. 1:**
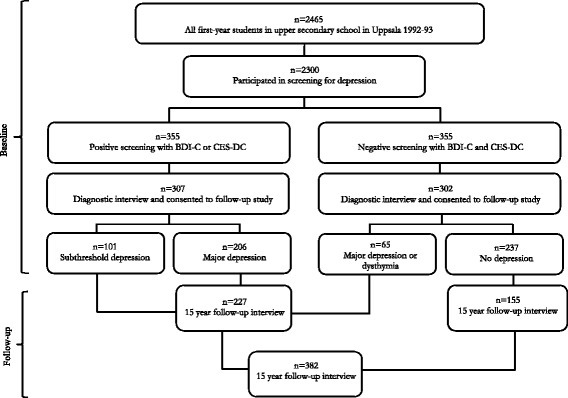
Chart outlining the data collection procedure at baseline (in adolescence) and at follow-up (in adulthood)

The present analyses are based on the 409 participants who participated in the follow-up. Participants with no identified depressive disorder or elevated depressive symptoms in adolescence were grouped together, while participants with an identified depressive disorder or elevated depressive symptoms were grouped together. The diagnostic interview in adolescence identified a previous depressive disorder before age 16 in a total of 44 of the non-depressed controls that were followed up, and these controls were accordingly transferred to the depression group. Participants with mania or hypomania in adolescence (*n* = 27) were excluded from the analyses because the etiology and mental health trajectory of bipolar disorder can differ from that of depressive disorders. Thus, the present study included 382 individuals; more specifically 227 individuals with prior depression and 155 non-depressed controls without prior depression or depressive symptoms. Figure 1 provides a description of the data collection procedure at baseline and at follow-up.

### Variables

The dependent variable was *major depression in adulthood*, based on information gathered at the follow-up diagnostic interview through M.I.N.I. [32]. Major depression was a dichotomous measure indicating whether or not the individual had suffered from one or more major depressive episodes between age 19 and approximately 31. We also conducted additional analyses of a number of other DSM-IV mental disorders assessed at follow-up through M.I.N.I.: *bipolar disorder, anxiety disorder*, *somatoform disorder*, *alcohol abuse*, *drug abuse*, and *psychotic episodes* (referring to the period from age 19 and until approximately 31, with the exceptions of somatoform disorders and some anxiety disorders which only covered the time at the follow-up).

The independent variables were constructed from information collected through the Children’s Life Events Inventory [30] in the baseline investigation. The main independent variable of interest was *parental separation*, based on questions asking whether the parents had moved apart and whether the parents had divorced. For both questions, the respondents were asked to reply whether this had happened during the past year or earlier in life. Participants who had reported that parents had moved apart or divorced (at any time point) were coded as having separated parents. Potential covariates that were included were *major conflicts between parents; major conflicts with parents; family income reduced considerably;* and *family moved to another city.* For each of these life events as well, participants were asked whether it had happened during the past year or earlier in life. We combined the answers to create binary measures of whether or not each life event had happened at all. *Parental depression* was recorded from the follow-up interview, more specifically from a constructed standardized interview that recorded different psychiatric diagnoses in close relatives, reported by the person interviewed and was coded as at least one parent having suffered from depression.

We also assessed the significance of *parental remarriage*, based on information from the Children’s Life Events Inventory [30] (whether, among individuals with separated parents, the mother and/or the father had remarried or had a new live-in partner). *Long-term depression* (depression most of the last year) and *somatic symptoms* (≥5 somatic symptoms according to the Somatic Symptom Checklist Instrument [34] – which from our earlier studies were shown to be important predictors of depression in adulthood [33, 35] – were included as markers of severity of the depression in adolescence.

### Statistical method

Chi-square tests were used to assess differences between groups. *T*-test was used to compare mean values. To adjust for potential confounders we conducted binary logistic regression models. To compare differences between individuals with and without separated parents, logit coefficients as well as odds ratios are presented. Since it is problematic to compare estimates from logistic regression analyses across models [36], as a sensitivity check we also conducted linear probability models (i.e. linear regression analyses of a dichotomous outcome), resulting in patterns similar to the ones presented (results available upon request).

## Results

### Descriptives

Descriptive statistics of the data are presented in Table 1, separately for the non-depressed control group and the depressed group (henceforth “non-depressed controls” and “depressed,” respectively). Differences between the two groups were assessed by chi-square tests. The shares of males and females were similar in both groups. Parental separation, major conflicts between parents and with parents, and having had a considerably reduced family income, were more commonly experienced by adolescents with depression than by the non-depressed controls. Parental depression was also more common among adolescents with depression. In adulthood, experiences of major depression were more common among individuals with adolescent depression than among non-depressed controls.

**Table 1:** Descriptive statistics. Percent (n in parentheses) and significance levels from chi-square tests

	Non-depressed controls (*n* = 155)	Depressed (*n* = 227)	*χ* ^2^ and *p*-value
*Baseline*			
Sex			
Male	20.6 (32)	20.7 (47)	
Female	79.4 (123)	79.3 (180)	0.00
Parental separation	27.1 (42)	41.4 (94)	8.23**
Major conflicts between parents	9.7 (15)	29.5 (67)	21.50***
Major conflicts with parents	5.2 (8)	27.3 (62)	30.20***
Family income considerably reduced	9.7 (15)	21.6 (49)	9.37**
Family moved to another city	29.0 (45)	35.2 (80)	1.61
Parental depression^a, b^	25.8 (40)	37.5 (84)	5.69*
*Follow-up*			
Major depression in adulthood	31.0 (48)	59.5 (135)	29.99***

***p < 0.001 **p < 0.01 *p < 0.05

^a^Non-depressed controls *n* = 155; depressed *n* = 224

^b^Question posed at follow-up but is retrospective

Next, the covariates at baseline and depression in adulthood among non-depressed controls and depressed respectively, are demonstrated by parental separation in childhood (Table 2). Among both non-depressed controls and depressed, major conflicts between parents in childhood were significantly more frequent among those with separated parents. Major conflicts with parents in childhood were also more frequent among those with separated parents than among those whose parents were not separated, although the difference was statistically significant only in the depressed group. Among both non-depressed controls and depressed, those with separated parents to a greater extent reported that their family income had been considerably reduced, compared with participants whose parents were not separated. Neither among non-depressed controls nor among depressed were there any significant differences in family moved to another city or in parental depression by parental separation. With regard to depression in adulthood, the prevalence among non-depressed controls did not differ by parental separation. Among participants who had suffered from adolescent depression, however, adult depression was significantly more common among those with separated parents in childhood (68.1%) than among those whose parents had not separated (53.4%). We also checked whether the severity of depression in adolescence, as indicated by long-term depression and by somatic symptoms, differed by parental separation among the individuals in the depressed group. This did not turn out to be the case, as 39.9% of those with non-separated parents and 40.4% of those with separated parents suffered from long-term depression (*p* = 0.931); and those with non-separated parents had on average 2.87 somatic symptoms compared with 2.97 symptoms among those with separated parents (*p* = 0.774) (data not shown).

**Table 2:** Covariates among non-depressed controls and depressed at baseline and depression at follow-up by parental separation. Percent and significance levels from chi-square tests

	Non-depressed controls (*n* = 155)			Depressed (*n* = 227)		
	Parents not separated	Parents separated	*χ* ^2^ and *p*-value	Parents not separated	Parents separated	*χ* ^2^ and *p*-value
*Baseline*						
Major conflicts between parents	3.5	26.2	17.97***	18.8	44.7	17.74***
Major conflicts with parents	4.4	7.1	0.46	21.8	35.1	4.91*
Family income considerably reduced	6.2	19.1	5.79*	15.8	29.8	6.38*
Family moved to another city	25.7	38.1	2.30	30.1	42.6	3.76
Parental depression^a, b^	22.1	35.7	2.95	33.3	43.5	2.38
*Follow-up*						
Major depression in adulthood	31.9	28.6	0.15	53.4	68.1	4.94*

***p < 0.001 *p < 0.05

^a^Non-depressed controls *n* = 155; depressed *n* = 224

^b^Question posed at follow-up but is retrospective

### Parental separation and depression at baseline

In Table 3, associations between parental separation and adolescent depression (i.e., depression at baseline) are presented. Logit coefficients and their standard errors, p-values, and odds ratios with 95% confidence intervals from binary logistic regressions are displayed, with “group” as the dependent variable (0 = non-depressed controls; 1 = depressed). Model 1 shows an excess risk of adolescent depression for those with separated parents (OR = 1.90, *p* = 0.004). When including potential covariates, it is seen that major conflicts between parents account for a rather substantial part of the association (Model 2). The association is attenuated somewhat also when including major conflicts with parents (Model 3), family income considerably reduced (Model 4), family moved to another city (although to a very minor extent) (Model 5), and parental depression (Model 6). When including all the potential covariates simultaneously (Model 7), the estimate of parental separation is attenuated and non-significant (OR 1.22, *p* = 0.428). All the included covariates except for family moves were significantly associated with depression at baseline (Models 2–6) but in the fully adjusted model (Model 7) only major conflicts between and with parents remained statistically significant. While the baseline investigation does provide information on whether the separation occurred during the past year or earlier in life, the limited number of cases in the former category prevents any meaningful analyses of time since parental separation. For adolescents whose parents separated last year (*n* = 13), we cannot rule out that some adolescent depression with very short duration may partly capture reactions of grief. Accordingly, we conducted sensitivity analyses where we excluded these individuals. The results were similar to the ones presented but with somewhat attenuated estimates for parental separation in all models (data not shown).

**Table 3:** Results from binary logistic regression analyses of parental separation in childhood and adolescent depression at baseline. Models adjusted for sex. *n* = 382

		coef.	s.e.		*p*	OR	95% CI	Pseudo R^2^
Model 1	Parental separation	0.64	0.23		0.004**	**1.90**	**1.22–2.96**	0.02
Model 2	Parental separation	0.36	0.24		0.128	1.44	0.90–2.30	0.05
	Major conflicts between parents	1.24	0.32	**<**	0.001***	**3.45**	**1.85–6.44**	
Model 3	Parental separation	0.50	0.24		0.034*	**1.65**	**1.04–2.61**	0.08
	Major conflicts with parents	1.86	0.39	**<**	0.001***	**6.41**	**2.96–13.88**	
Model 4	Parental separation	0.54	0.23		0.019*	**1.72**	**1.09–2.69**	0.03
	Family income reduced	0.83	0.32		0.010*	**2.29**	**1.22–4.29**	
Model 5	Parental separation	0.62	0.23		0.007**	**1.85**	**1.19–2.89**	0.02
	Family moved to another city	0.21	0.23		0.365	1.23	0.79–1.93	
Model 6^a^	Parental separation	0.57	0.23		0.012*	**1.78**	**1.14–2.78**	0.02
	Parental depression	0.48	0.23		0.039*	**1.62**	**1.02–2.56**	
Model 7^a^	Parental separation	0.20	0.25		0.428	1.22	0.74–2.02	0.10
	Major conflicts between parents	0.76	0.35		0.030*	**2.13**	**1.08–4.24**	
	Major conflicts with parents	1.54	0.41	**<**	0.001***	**4.64**	**2.08–10.36**	
	Family income reduced	0.56	0.34		0.102	1.75	0.89–3.44	
	Family moved to another city	0.05	0.25		0.831	1.05	0.65–1.71	
	Parental depression	0.32	0.25		0.191	1.38	0.85–2.25	

***p < 0.001 **p < 0.01 *p < 0.05

^a^ *n* = 379

Bold text indicates results with *p*-values < 0.05

### Parental separation and depression at follow-up

Results from the analyses of the associations between parental separation in childhood and depression in adulthood are displayed in Table 4. Among the non-depressed controls, there was no significant association between parental divorce and adult depression (Model 1) (reflecting the descriptive statistics in Table 2) and the estimates did not change considerably when the covariates were included (Models 2–6). Among individuals with adolescent depression, however, there was an excess risk of adult depression among those whose parents were separated compared with those whose parents were not (Model 1, OR = 1.99, *p* = 0.016). The association was not accounted for more than marginally by any of the potential covariates (Models 2–6). When adjusting simultaneously for all the potential covariates (Model 7), the odds ratio was attenuated and reached statistical significance only at the 10% level (OR = 1.74, *p* = 0.081). Again, we performed sensitivity analyses where we excluded the 13 individuals whose parents separated the year before the baseline investigation, with results very similar to the ones demonstrated in Table 4, with no visible attenuation (data not shown). Additional analyses of individuals with adolescent depression and separated parents (not shown) tested whether parental remarriage was associated with depression in adulthood. Results indicated a tendency for parental remarriage to be associated with a decreased risk of depression, although this was statistically significant at the 10% level only (OR = 0.41, *p* = 0.092).

**Table 4:** Results from binary logistic regression analyses of parental separation in childhood and major depression in adulthood. Models adjusted for sex

		coef.	s.e.	*p*	OR	95% CI	Pseudo R^2^
Non-depressed controls (*n* = 155)							
Model 1	Parental separation	−0.24	0.40	0.544	0.78	0.36–1.73	0.01
Model 2	Parental separation	−0.19	0.42	0.659	0.83	0.36–1.90	0.01
	Major conflicts between parents	−0.27	0.65	0.679	0.76	0.21–2.73	
Model 3	Parental separation	−0.24	0.40	0.558	0.79	0.36–1.74	0.01
	Major conflicts with parents	−0.34	0.84	0.688	0.71	0.14–3.70	
Model 4	Parental separation	−0.23	0.41	0.582	0.80	0.35–1.79	0.01
	Family income reduced	−0.12	0.63	0.845	0.88	0.26–3.04	
Model 5	Parental separation	−0.27	0.41	0.501	0.76	0.34–1.69	0.01
	Family moved to another city	0.20	0.39	0.596	1.23	0.58–2.61	
Model 6^a^	Parental separation	−0.24	0.41	0.551	0.78	0.35–1.74	0.01
	Parental depression	−0.02	0.41	0.968	0.98	0.44–2.18	
Model 7^a^	Parental separation	−0.19	0.44	0.664	0.83	0.35–1.95	0.02
	Major conflicts between parents	−0.35	0.70	0.616	0.70	0.18–2.80	
	Major conflicts with parents	−0.42	0.87	0.625	0.65	0.12–3.58	
	Family income reduced	−0.02	0.66	0.977	0.98	0.27–3.61	
	Family moved to another city	0.31	0.41	0.453	1.36	0.61–3.04	
	Parental depression	−0.09	0.42	0.832	0.91	0.40–2.09	
Depressed (*n* = 227)							
Model 1	Parental separation	0.69	0.29	0.016*	**1.99**	**1.13–3.49**	0.03
Model 2	Parental separation	0.64	0.30	0.032*	**1.89**	**1.05–3.38**	0.03
	Major conflicts between parents	0.21	0.32	0.513	1.23	0.66–2.30	
Model 3	Parental separation	0.61	0.29	0.035*	**1.85**	**1.05–3.27**	0.04
	Major conflicts with parents	0.64	0.33	0.052	1.89	0.99–3.60	
Model 4	Parental separation	0.65	0.29	0.026*	**1.91**	**1.08–3.37**	0.03
	Family income reduced	0.35	0.35	0.315	1.42	0.71–2.84	
Model 5	Parental separation	0.62	0.29	0.032*	**1.86**	**1.05–3.29**	0.04
	Family moved to another city	0.67	0.30	0.026*	**1.96**	**1.08–3.54**	
Model 6^a^	Parental separation	0.69	0.30	0.020*	**2.00**	**1.12–3.57**	0.05
	Parental depression	0.67	0.30	0.026*	**1.95**	**1.08–3.53**	
Model 7^a^	Parental separation	0.55	0.32	0.081	1.74	0.93–3.23	0.08
	Major conflicts between parents	−0.20	0.37	0.594	0.82	0.40–1.70	
	Major conflicts with parents	0.72	0.38	0.057	2.06	0.98–4.34	
	Family income reduced	0.09	0.37	0.800	1.10	0.53–2.28	
	Family moved to another city	0.75	0.32	0.019*	**2.12**	**1.13–3.97**	
	Parental depression	0.60	0.31	0.055	1.82	0.99–3.35	

*p < 0.05

^a^Non-depressed controls *n* = 155; depressed *n* = 224

Bold text indicates results with *p*-values < 0.05

The purpose of the study was to assess whether there was an association between parental separation and depression in adulthood *within* the two studied groups, i.e., among the depressed and among the non-depressed controls, respectively. The analyses in Table 4 showed that parental separation was associated with depression in adulthood among the depressed but not among the non-depressed controls. In order to assess whether the association between parental separation and depression in adulthood also differed *between* these two groups, additional analyses (not shown) were performed. We performed logistic regression analyses of the pooled sample (i.e., the depressed and the non-depressed merged together) and tested for the interaction between adolescent depression and parental separation. The interaction term was borderline significant at the 5% level (*p* = 0.052) (data not shown).

### Parental separation and other mental disorders at follow-up

It is possible that the experience of parental separation predicts other mental illnesses than depression. Accordingly, we conducted additional analyses of parental separation in childhood and bipolar disorder, anxiety disorder, somatoform disorder, alcohol and substance abuse as well as the occurrence of psychotic episodes in adulthood, among non-depressed controls and depressed, respectively (Table 5). The only detected statistically significant association indicated that among individuals who had suffered from depression in adolescence, there was an excess risk for bipolar disorders in adulthood (OR 2.37, *p* = 0.048). The association was slightly attenuated and turned non-significant when adjusting for all the potential confounders (OR 2.18, *p* = 0.103) (data not shown). In addition, a separate analysis (not shown) of the non-depressed controls who suffered from anxiety disorder in childhood or adolescence showed that parental separation was associated with a statistically significant excess risk of continuation of anxiety in adulthood. In this particular subgroup, 14.3% of those with non-separated parents and 60.0% of those with separated parents met the criteria for an anxiety disorder in adulthood (*p* = 0.019). Among adolescents with depression and anxiety, parental separation was not significantly associated with anxiety in adulthood (*p* = 0.335) (data not shown).

**Table 5:** Results from binary logistic regression analyses of parental separation in childhood and various DSM-IV mental disorders in adulthood. Models adjusted for sex

	coef.	s.e.	*p*	OR	95% CI	Pseudo R^2^
Non-depressed controls (*n* = 155)						
Bipolar disorder	0.45	1.27	0.725	1.57	0.13–19.00	0.01
Anxiety disorder	0.23	0.43	0.590	1.26	0.54–2.91	0.02
Somatoform disorder	2.33	1.22	0.055	10.30	0.95–111.58	0.12
Alcohol abuse	−0.30	0.83	0.713	0.74	0.15–3.72	0.05
Drug abuse^a^	-	-	-	-	-	-
Psychotic episodes^b^	1.00	1.43	0.481	2.73	0.17–44.69	0.02
Depressed (*n* = 227)						
Bipolar disorder	0.86	0.44	0.048*	**2.37**	**1.01–5.55**	0.03
Anxiety disorder	0.01	0.27	0.964	1.01	0.59–1.73	0.01
Somatoform disorder	0.67	0.42	0.108	1.95	0.86–4.41	0.02
Alcohol abuse	−0.08	0.40	0.836	0.92	0.42–2.02	0.00
Drug abuse	−0.13	0.52	0.811	0.88	0.32–2.45	0.03
Psychotic episodes^b^	−0.36	0.72	0.617	0.70	0.17–2.86	0.00

*p < 0.05

^a^Analyses of non-depressed controls not possible due to empty cells

^b^Analyses of non-depressed controls and depressed not adjusted for sex due to empty cells

Bold text indicates results with *p*-values < 0.05

## Discussion

The aim of this study was to investigate whether parental separation in childhood was associated with major depression in adulthood. In order to assess whether such an association was particularly prominent among individuals who had suffered from depression in adolescence, we utilized data from a community-based study of adolescents with depression and non-depressed controls followed prospectively 15 years after baseline. The results showed that parental separation was not associated with an excess risk of depressive disorder among the non-depressed controls. However, among individuals who had suffered from depression in adolescence, parental separation was linked with an excess risk of recurrence of depression in adulthood, albeit with a moderate effect size. Thus, while the negative effects of parental divorce are relatively small on average [4], the present study shows that they may be greater for specific vulnerable groups and smaller or even non-existent for others.

While earlier studies of Swedish data found that, overall, parental divorce was not associated with later mental illness [12] or adult psychiatric care [11], the present study adds to the previous literature by assessing whether the “effect” of parental separation may differ between groups, as has been suggested [13]. The association between parental separation and depression in adulthood in this vulnerable group was only to a limited extent accounted for by potential covariates: conflicts between and with parents, that the family’s income had been considerably reduced, that the family had moved to another city, or that one or both parents had suffered from depression. Thus, there are probably other mechanisms that we were not able to include in the present study. We found that inter-parental conflicts accounted for part of the association between parental separation and adolescent depression at baseline, but this did not contribute much to explaining the association between parental separation and depression in adulthood. This is in contrast to the findings reported by Gähler and Palmtag [18], who showed that the poorer mental health found among adults whose parents had divorced during their childhood was accounted for by economic difficulties and greater family dissension. A possible explanation of why our results differ from those of Gähler and Palmtag [18] may relate to the different designs of the data materials used. While the present study used a community-based sample of adolescents with depression and matched non-depressed peers who were followed-up prospectively and assessed with clinical mental health diagnoses, Gähler and Palmtag’s study was based on a representative sample of the adult population in Sweden, using retrospective data (implying the risk of recall bias), and with a self-reported mental health measure. Thus, it is possible that parental conflict accounts for the association between parental separation and less severe mental health problems but in case of major depression there seem to be also other mechanisms at work.

What, then, are the underlying reasons for the excess risk of depression in adulthood among individuals who had suffered from depression in adolescence and whose parents were divorced? One possible explanation may be that living in a single-parent family (which the majority of the individuals with separated parents had likely done for a shorter or longer period) often entails limited resources, both in terms of poorer socioeconomic resources but also with regard to less provision of social support and monitoring, which, in turn, are likely to be linked to poorer mental health outcomes among offspring. The finding that parental remarriage possibly seemed to be protective of relapse into depression (although only at the 10% level, data not shown) supports the interpretation that conditions associated with single parenthood may drive the associations between parental separation and later depression in the subgroup of depressed adolescents. The elevated risk of depression in adulthood among individuals with adolescent depression and separated parents could also potentially be an effect of a more severe adolescent depression in this subgroup. However, neither long depression nor somatic symptoms differed between these two subgroups, thus indicating that the adolescent depression was not more severe among individuals with separated parents than among those with non-separated parents. Other possible pathways in the association between parental separation and relapse into depression may include inflated self-concept and problematic interpersonal coping strategies. Assessing such potential mechanisms is a task for future research.

An incidental finding was that among individuals with adolescent depression, parental separation was linked with an excess risk not only of major depression but also of bipolar disorder in adulthood. This implies that parental separation seems to be associated with increased risk of chronicity of disorders already present in the individual, as well as with an increased risk of switching to a bipolar disorder. Future research is however needed to confirm this finding. Previous analyses of the same cohort have shown that a family history of bipolar disorder was a strong significant risk factor for bipolar disorder in adulthood [37]. The number of individuals in the data material with bipolar disorder in adulthood is however too small to disentangle relationships between parents’ bipolar disorder, parental separation, and the individual’s depression and bipolar disorder. Consequently, exploring the possible mechanisms using a larger data material is a promising task for future research.

Furthermore, the interpretation that parental separation is linked to a risk of chronicity in illnesses already present in the individual is supported by our analyses of anxiety disorder, which showed that among non-depressed controls with anxiety disorder in childhood or adolescence according to DICA-R-A, parental separation was linked with an excess risk of recurrence of anxiety in adulthood.

### Strengths and limitations

The data used have several strengths. The data collected at baseline were community-based and included 2,300 adolescents of the same age with a high participation rate in the depression screening (93%). The non-depressed controls were matched to the depressed by sex, age, and school class. The data included clinical interview diagnoses both at baseline and at follow-up. One limitation is that only about two thirds of participants in the original investigation also took part in the follow-up. Still, the participation rate can be seen as reasonably high in relation to the follow-up period of 15 years. Furthermore, the attrition was evenly distributed between the non-depressed control and the depressed group. In addition, Jonsson et al. [33], using the same data, concluded that the studied baseline characteristics did not indicate that the attrition between baseline and follow-up resulted in biased findings. Their analyses using a multiple imputation approach showed overall similar results to those from analyses containing only complete cases. Nevertheless, Jonsson et al. [33] did not rule out the possibility that there could be bias due to other factors. Another limitation is that we lack proper measures of socioeconomic conditions in childhood. We also lack information on the time point of the parental separation, which may be relevant to include as a measure of time spent in a non-nuclear family. As recently highlighted in a review, earlier research has not been able to identify that experiencing parental separation at a particular age or development stage is especially critical for developing later depression [14]. Higher distress scores have been recorded for those who experienced separation at younger ages (0–16 years) than at older ages (17–33 years) [38] but it has also been shown that father absence in early childhood (child <5 years of age) predicted self-reported depressive symptoms at age 14, whereas father absence in middle childhood (child ≥5 years of age) did not [39]. As a sensitivity check, we performed additional analyses where we excluded individuals whose parents had separated the year before the baseline investigation. For the cross-sectional analyses of baseline data, it was shown that the “effect” of parental divorce on concurrent depression was somewhat weaker than in the analyses which included these individuals. For the prospective analyses of depression in adulthood, the results were however very similar irrespective of whether or not these individuals were included. Accordingly, our crude measure of the timing of parental separation did not seem to have a moderating role for the risk of depression in adulthood. Nevertheless, information on the timing of separation would indeed have been valuable in order to better distinguish between our covariates as confounders or as mediators. We believe this is important to consider in future inquiry about the links between parental divorce and later depression in different subgroups. Another limitation is the increased risk of type I errors stemming from multiple comparisons. However, we still judge that the general pattern of results is valid. Finally, even though we used community-based data, the generalizability of our findings to other populations than the one investigated is not straightforward and thus further studies are needed to corroborate the results in other contexts.

## Conclusions

Adolescent depression appears to be a moderator in the association between parental separation and adult depression. Among depressed adolescents, parental separation seems to predict relapse into depression in adulthood. By contrast, among non-depressed adolescents, parental separation does not appear to be associated with an excess risk of suffering from future depression.

As a consequence, among adolescents with major depression, attention should be paid to those who also have separated parents. These adolescents in particular might benefit from qualified treatment and longer follow-up periods. Additionally to standard treatment like antidepressant medication and cognitive behavior therapy other treatment and supportive strategies might be added, for instance, family interventions and, if needed, cooperation with the social services. For example, previous studies have shown that parental sensitivity can be strengthened and work as a buffer against the risk of future depressive episodes among children [14].
